# Reexamining empathy in autism: Empathic disequilibrium as a novel predictor of autism diagnosis and autistic traits

**DOI:** 10.1002/aur.2794

**Published:** 2022-08-20

**Authors:** Ido Shalev, Varun Warrier, David M. Greenberg, Paula Smith, Carrie Allison, Simon Baron‐Cohen, Alal Eran, Florina Uzefovsky

**Affiliations:** ^1^ Psychology Department Ben Gurion University of the Negev Beer‐Sheba Israel; ^2^ Zlotowski Center for Neuroscience Ben Gurion University of the Negev Beer‐Sheba Israel; ^3^ Department of Psychiatry, Autism Research Centre University of Cambridge Cambridge UK; ^4^ Interdisciplinary Department of Social Sciences and Department of Music Bar‐Ilan University Ramat Gan Israel; ^5^ Life Sciences Department Ben Gurion University of the Negev Beer‐Sheba Israel; ^6^ Computational Health Informatics Program Boston Children's Hospital Boston Massachusetts USA

**Keywords:** cognitive, empathy, autism, emotional empathy, empathy, response surface analysis

## Abstract

A large body of research showed that autistic people have intact emotional (affective) empathy alongside reduced cognitive empathy. However, there are mixed findings and these call for a more subtle understanding of empathy in autism. Empathic disequilibrium refers to the imbalance between emotional and cognitive empathy and is associated with a higher number of autistic traits in the typical population. Here we examined whether empathic disequilibrium predicts both the number of autistic traits and autism diagnosis. In a large sample of autistic (*N* = 1905) and typical individuals (*N* = 3009), we examined empathic disequilibrium and empathy as predictors of autistic traits and autism diagnosis, using a polynomial regression with response surface analysis. Empathy and autistic traits were measured using validated self‐report questionnaires. Both empathic disequilibrium and empathy predicted linearly and non‐linearly autism diagnosis and autistic traits. Specifically, a tendency towards higher emotional than cognitive empathy (empathic disequilibrium towards emotional empathy) predicted both autism diagnosis and the social domain of autistic traits, while higher cognitive than emotional empathy was associated with the non‐social domain of autism. Empathic disequilibrium was also more prominent in autistic females. This study provides evidence that beyond empathy as was measured thus far, empathic disequilibrium offers a novel analytical approach for examining the role of empathy. Empathic disequilibrium allows for a more nuanced understanding of the links between empathy and autism.

**Lay summary:**

Many autistic individuals report feelings of excessive empathy, yet their experience is not reflected by most of the current literature, typically suggesting that autism is characterized by intact emotional and reduced cognitive empathy. To fill this gap, we looked at both ends of the imbalance between these components, termed empathic disequilibrium. We show that, like empathy, empathic disequilibrium is related to autism diagnosis and traits, and thus may provide a more nuanced understanding of empathy and its link with autism.

## BACKGROUND

Autism is a common neurodevelopmental condition and is characterized by marked social difficulties, especially in communication (American Psychiatric Association, 2013). The difficulty in communicating may partially explain why many autistic individuals are vulnerable to exclusion, show high rates of mental health difficulties including depression and anxiety, and often report feeling misunderstood by others (Camm‐Crosbie et al., 2019; Lever & Geurts, 2016; Maddox et al., 2017).

These difficulties in communication have often been studied as reflecting empathy deficits (Decety & Moriguchi, 2007); yet such conceptualization is sometimes at odds with autistic individuals' accounts (Gillespie‐Lynch et al., 2017). Therefore, there is a need to understand empathy in autism. Many studies show that autistic individuals are characterized by deficits in cognitive empathy (recognizing another person's mental states) alongside intact emotional (or affective) empathy (responding to another person's mental states with an appropriate emotion) (Baron‐Cohen, 2013). Other studies find that some autistic individuals show typical cognitive empathy and some even report an excess of emotional empathy (Gillespie‐Lynch et al., 2017; Lombardo et al., 2016). This suggests that ‘deficits’ in empathy do not accurately reflect autistic individuals' empathy abilities. It is therefore important to examine how the two empathy components interact. The concept of empathic disequilibrium, that is, the tendency to experience relatively higher emotional than cognitive empathy (or vice versa) reflects the relationship between the two components, and is associated with autistic traits in the typical population, above and beyond overall empathy (Shalev & Uzefovsky, 2020). In the current study, we examined whether empathic disequilibrium is also associated with an autism diagnosis.

Empathy is the ability to understand another's mental states and respond to these with an appropriate emotion (Decety & Jackson, 2004). Empathy can be separated into an emotional and a cognitive component. Emotional empathy (EE), also called affective empathy, is the ability to respond to another's mental state with an appropriate emotion, and cognitive empathy (CE) is the ability to recognize what another person is thinking or feeling (Baron‐Cohen & Wheelwright, 2004).

The two empathy components are rooted in distinct yet interrelated neurobiological evolved mechanisms and have different developmental, neural, and genetic trajectories. From a developmental perspective, EE appears very early in life and remains stable or increases slightly during the second year of life (Knafo et al., 2008). Conversely, CE develops later in the first year of life and increases throughout life.

A recent meta‐analysis of twin studies (*k* = 23; Abramson et al., 2020) revealed that the dissociation between EE and CE is also supported at the genetic level with EE more influenced by heritability than CE (estimated 48.3% for EE and 26.9% for CE), and CE, unlike EE, depending on shared‐environment (Abramson et al. suggested that cultural norms and beliefs about emotions were possible shred‐environmental factors). There is some indication that the difference in heritability is subserved by different genes related to each trait (Pearce et al., 2017; Uzefovsky et al., 2014; Uzefovsky et al., 2015).

Further support is provided by neuroimaging studies showing that CE and EE differ in brain activation and structure (de Waal & Preston, 2017; Shamay‐Tsoory et al., 2008; Uribe et al., 2019). CE depends on neocortical brain regions such as the ventromedial prefrontal cortex, whereas EE depends on subcortical regions such as the amygdala and the anterior cingulate cortex.

Of note, while most studies have focused on distinguishing the two traits, CE and EE‐related brain regions partially overlap, and regions specifically associated with each trait were suggested to interact when additional information is needed to engage with the feelings of others (Lamm et al., 2011; Preckel et al., 2018). For example, CE‐related brain regions interact with EE‐related brain regions, particularly during complex social situations in which additional processing is needed to jointly engage EE and CE (Lamm et al., 2011). As we constantly encounter complex social situations, these studies suggest that maintaining a balance between EE and CE is key for an adaptive and appropriate social response, leading to effective social communication.

Consistently, autistic individuals, which show marked difficulties in social communication, are usually reported as having intact EE but impaired CE (Baron‐Cohen, 2013). Although this finding is supported by many empirical pieces of evidence (Gleichgerrcht et al., 2013; Mul et al., 2018; Rueda et al., 2015), other studies show mixed results (aan het Rot & Hogenelst, 2014; Mazza et al., 2014; Scambler et al., 2007). For example, one study found that young autistic children displayed EE less frequently than non‐autistic children (Scambler et al., 2007). Another study used a common behavioral task to measure CE and classified autistic individuals into five subgroups, two of which did not differ in CE from non‐autistic individuals (Lombardo et al., 2016). These mixed findings suggest that the conceptualization of the social difficulties in autism as stemming from a deficit in CE, without the involvement of EE, is not quite accurate. Additionally, most previous studies of empathy in autism have examined the role of CE and EE independently, overlooking the possible interdependence between the two traits.

Trying to bridge this gap, we recently showed that empathic disequilibrium that is, the level of imbalance between CE and EE, predicted autistic traits in typical individuals, even after controlling for their total empathy (Shalev & Uzefovsky, 2020). Specifically, we showed that autistic traits were elevated in a group of individuals with relatively higher EE than CE (EE‐dominance group) and found that EE‐dominance was related to social traits relevant to autism, such as alexithymia; but not to the non‐social traits, such as systemizing. These findings provide empirical evidence for the notion that an imbalance between CE and EE might contribute to some autistic symptoms (Smith, 2009).

**FIGURE 1:**
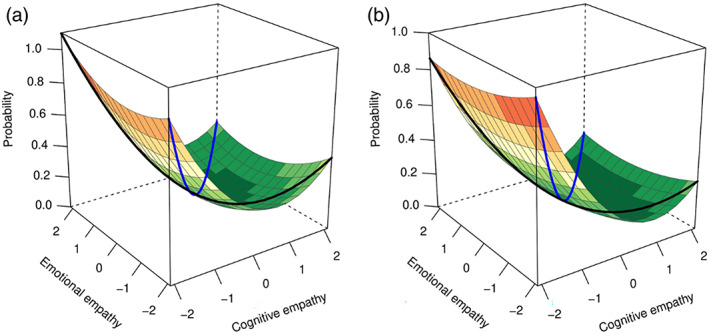
Polynomial regression plot predicting the probability of autism diagnosis. A plot of the polynomial regression with response surface analysis of emotional and cognitive empathy, predicting the probability of autism diagnosis. The black line represents empathic disequilibrium, and the blue line represents total empathy. (a) The response surface parameters for males (*N* = 1641), (b) for females (*N* = 3273)

The current study aimed to examine the role of empathic disequilibrium in a large sample of clinically diagnosed autistic individuals to establish its relevance to autism. In our previous analysis, we used difference scores to compute empathic disequilibrium (Shalev & Uzefovsky, 2020), yet such an approach suffers from some problems (see Edwards, 1994, 1995 for a comprehensive review). Difference scores tend to be less reliable than each component separately, introducing possible bias to the results (Cronbach & Furby, 1970). Moreover, by reducing the dimensionality of the two components, difference scores limit the interpretation of the results, making it harder to tell, in the current case, whether it was EE‐dominance or CE‐dominance that contributed to the prediction of autistic traits. To overcome these limitations, we now utilize polynomial regression with response surface analyses, providing a more nuanced and statistically valid methodology to examine the imbalance between CE and EE (Edwards & Parry, 1993; Shanock et al., 2010). Polynomial regression with response surface analyses allows one to visualize and estimate the line of congruence, representing the degree to which similarity between variables is associated, both linearly and curvilinearly, with an outcome variable (see the blue line in Figure 1); and the line of incongruence, which examines whether and how the discrepancy between two variables is related to an outcome (see the black line in Figure 1). In this case, the line of incongruence represents empathic disequilibrium, and the line of congruence represents total empathy (the combination of EE and CE). Thus, we examine whether equilibrium and disequilibrium between EE and CE predict autism diagnosis and autistic traits (social and non‐social aspects).

Based on our previous findings (Shalev & Uzefovsky, 2020), we hypothesized that both total empathy and empathic disequilibrium (with higher EE than CE) would predict autism and social traits related to autism. In contrast, empathic disequilibrium with higher CE than EE will predict non‐social traits related to autism (e.g., systemizing). Furthermore, as empathic disequilibrium also shows sex differences, we expected females to show a higher tendency towards EE‐dominance, relative to males, on average and across diagnostic groups.

## METHODS

All participants signed informed consent before participation.

### Participants

Participants were 2076 individuals diagnosed with autism and 3494 non‐autistic controls recruited via the Cambridge Autism Research Database between 2003–2020. Of those, 486 participants did not complete the empathy measure and were excluded. Additional 169 participants were excluded before data analysis as they were under the age of 18. After exclusion, participants were 1905 individuals diagnosed with autism (54% females, *M*_age_ = 36.81 ± 12.88, range 18–80 years) and 3009 typical controls (75% females, *M*_age_ = 38.26 ± 12.31 range 18–92 years; see Table 1). Participants self‐reported their diagnosis (including specific details, such as date of diagnosis, which is used as a validity check for diagnostic status), age, and birth sex. Autistic and typical participants then completed a battery of questionnaires. All questionnaires were filled online, suggesting most participants showed at least near‐normal intelligence. The typical group showed elevated (yet in the typical range of) autistic traits, and 193 individuals exceeded the autism cut‐off of 32 on the Autism‐Spectrum Quotient (Baron‐Cohen et al., 2001), suggesting that this group, although undiagnosed, shows elevated features of autism.

**TABLE 1:** Descriptive statistics

	Non‐autistic		Autism	
	Females	Males	Females	Males
*N*	2246	763	1027	878
Age	38.5 (11.5)	37.6 (14.5)	35.6 (11.9)	38.2 (13.9)
Autism quotient	17.1 (8)	19.8 (8.3)	39.2 (6.45)	37.9 (7.2)
Systemizing quotient	53.4 (20.8)	68 (22.4)	77.4 (24.2)	81.6 (25)
Overall empathy	49.6 (14.2)	39.5 (14.7)	19.9 (10.6)	17.4 (10.6)
Emotional empathy	6.57 (2.37)	4.89 (2.53)	3.65 (2.69)	2.91 (2.46)
Cognitive empathy	6.47 (2.66)	5.03 (2.92)	1.51 (2.06)	1.25 (1.96)

*Note*: Descriptive statistics comparing autistic to non‐autistic individuals, stratified by sex. Mean and standard deviation (in parenthesis) are reported.

### Measures

#### Empathy

Empathy was measured using the Empathy Quotient (EQ; Baron‐Cohen & Wheelwright, 2004). This questionnaire consists of 60 items (40 empathy items and 20 filler items) on a four‐point scale. On each empathy item, a person can score 2, 1, or 0. We calculated EE and CE following Muncer and Ling's classification (Muncer & Ling, 2006) (see Table S1). We did not include the ‘social skills’ subscale as it does not directly relate to EE and CE. Using this classification, EE and CE were positively correlated (*r* = 0.59, *p* < 1x10^−100^). Following Fleenor et al. (1996) recommendation, in line with our previous study (Shalev & Uzefovsky, 2020), and in the aim of creating an easily interpretable measure, we standardized EE and CE (separately) as follows. We divided each measure by the standard deviation of the sample and centered them based on the mean of the control group. Thus, the scores reflect the standardized score of CE and EE relative to the mean of the non‐autistic population.

#### Autistic traits

Autistic traits were measured using the Autism‐Spectrum Quotient (AQ; Baron‐Cohen et al., 2001). This questionnaire consists of 50‐items measuring autistic traits. Responses are scored using a binary system, where an endorsement of the autistic trait (either mildly or strongly) is scored as 1, while the opposite response is scored as 0. Scores are then summed up leading to a maximum score on the AQ of 50. The AQ can also be divided according to five domains: ‘social ‘skills', ‘attention ‘switching’, ‘attention to ‘detail’, “communication’, and “imagination’.

We also measured systemizing, which is the drive to analyze or construct systems and is a non‐social autism‐related trait. Systemizing was measured using the Systemizing Quotient (Baron‐Cohen et al., 2003), a 60 items (40 systemizing items and 20 filler items) questionnaire with a 0–2 rating scale, with higher scores representing higher systemizing.

### Statistical analyses

#### Sex differences analysis

In our previous analysis, empathic disequilibrium was found to be sex‐sensitive (Shalev & Uzefovsky, 2020). Therefore, we examined the mean empathic disequilibrium differences between males and females in autistic and non‐autistic participants. A 2 × 2 ANCOVA was conducted examining sex, diagnosis, and their interaction as predictors of empathic disequilibrium, controlling for age. As methods that predict parameters derived from the polynomial regression are not yet fully developed and validated (Edwards, 1995), we calculated empathic disequilibrium by subtracting standardized CE from standardized EE, as previously described (Shalev & Uzefovsky, 2020).

#### Difference score analysis of empathic disequilibrium and total empathy

As described earlier (see introduction), polynomial regression with response surface analyses holds many advantages over traditional analysis using difference scores (Edwards, 1994, 1995). However, using multiple regression with both empathic disequilibrium and total empathy within the same model has the advantage of allowing us to quantify the unique contribution of each predictor beyond the other. Such multiple regression analyses were conducted to predict autism diagnosis (with logistic regression) and traits (using AQ and SQ). The three‐ and two‐way interactions between empathic disequilibrium, sex, and diagnosis were also examined. Age was controlled for as a covariate.

#### Response surface analysis of empathy

To examine empathic disequilibrium and its relevance to autism, we applied polynomial regression with response surface analyses (Edwards & Parry, 1993; Shanock et al., 2010). The polynomial regression tested both the linear and curvilinear patterns of total empathy, defined as the line of congruence, and of empathic disequilibrium, defined as the line of incongruence, using a polynomial regression between EE and CE as described using the following Equation (1):

(1) $$Z=b_{0}+b_{1}\mathrm{CE}+b_{2}\mathrm{EE}+b_{3}{\mathrm{CE}}^{2}+b_{4}\mathrm{CE}\times \mathrm{EE}+b_{5}{\mathrm{EE}}^{2}+e.$$

To interpret the surface of the polynomial regression, regression coefficients are used to extract four surface parameters, as follows:

1. The linear association between total empathy and an outcome variable (*a*1 = *b*1 + *b*2).
2. The curvilinear association between total empathy and an outcome variable (*a*2 = *b*3 + *b*4 + *b*5).
3. The linear association between empathic disequilibrium and an outcome variable (*a*3 = *b*1 − *b*2).
4. The curvilinear association between empathic disequilibrium and an outcome variable (*a*4 = *b*3 − *b*4 + *b*5).

Therefore, *a*1 and *a*2 reflect the association between total empathy and an outcome, while *a*3 and *a*4 reflect the association between empathic disequilibrium and an outcome.

#### Autism prediction using polynomial regression

We first wanted to examine if the polynomial regression surface created using EE and CE and its derived total empathy (i.e., *a*1 and *a*2) and empathic disequilibrium (i.e., *a*3 and *a*4) parameters predict autism diagnosis. To do so, we conducted a polynomial logistic regression with autism diagnosis as a binary outcome. We also examined whether the surface parameters differed between the sexes. Due to the relatively large variability in age in this sample, it was included as a covariate. Excluding age as a covariate did not alter any of the findings (see Appendix S1).

#### Autistic traits prediction using polynomial regression

Using polynomial regression with response surface analyses, we also examined whether total empathy and empathic disequilibrium predicted autistic traits and whether surface parameters differed between autistic and typical individuals. To do so, we conducted a polynomial regression analysis using EE and CE for AQ and SQ as outcome variables (separately). Differences in surface parameters were investigated between autistic and non‐autistic individuals. Age and sex were used as covariates. We also conducted polynomial regression analyses for each of the five AQ subscales separately (see Appendix S1).

All analyses were carried out using R v4.0.3 ‘stats’ package (R Core Team, 2014). RSA plots were produced using the RSA package in R (Schönbrodt & Humberg, 2016).

## RESULTS

### Sex differences in empathic disequilibrium

**FIGURE 2:**
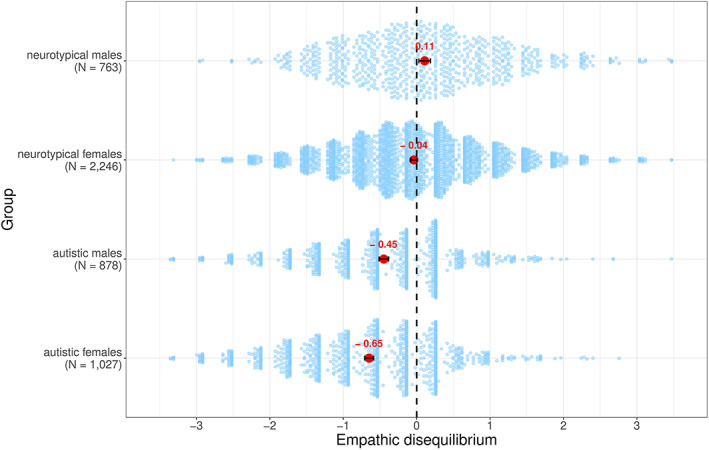
Sex and diagnosis differences in empathic disequilibrium. The mean of each group appears in red. 95% confidence intervals of each group are depicted. Positive values of empathic disequilibrium (on the *x*‐axis) represent higher cognitive than emotional empathy. Negative values represent higher emotional than cognitive empathy. The dashed line represents the point of no difference between cognitive and emotional empathy

An ANOVA revealed significant main effect for sex (*F*[1,4910] = 29.47, *p* = 6 × 10^−8^, *ƞ*_p_^2^ = 0.006) and diagnosis (*F*[1,4910] = 392.4, *p* = 5 × 10^−79^, *ƞ*_p_^2^ = 0.07). No effects were found for the interaction (*F*[1,4910] = 0.55, *p* = 0.46) or age (*F*[1,4910] = 0.12, *p* = 0.72). To better understand these results, we conducted post hoc one‐sample *t* test analyses to examine whether the mean of each group differs from a balanced empathy score (CE equals EE; empathic disequilibrium = 0). Autistic males and females differed significantly from equilibrium (*t*(877) = −13.54, *p* = 5 × 10^−38^ for males; *t*(1026) = −20.905, *p* = 4 × 10^−81^ for females) showing higher EE than CE. Typical males differed from equilibrium showing higher CE than EE (*t*(762) = 2.84, *p* = 0.005), while typical females did not differed from equilibrium (*t*(2245) = −1.76, *p* = 0.08). Results are shown in Figure 2.

### Difference score analysis of empathic disequilibrium and total empathy

Multiple regression analyses using empathic disequilibrium calculated as a difference score, revealed that empathic disequilibrium is a stronger predictor of autism diagnosis (OR = 0.63 [0.58–0.68, 95% CI], *p* < 1 × 10^−100^), than total empathy (OR = 0.87 [0.86–0.88, 95% CI], *p* < 1 × 10^−100^). Beyond total empathy, empathic disequilibrium also uniquely contributed to the prediction of social and non‐social autistic traits, although to a lesser extent. Full results for these analyses are described in the Appendix S1.

#### Response surface analysis of empathy

We next examined how total empathy and empathic disequilibrium predict autism diagnosis and autism‐related traits using polynomial regression models. Residuals of all the models tested were normally distributed.

#### Predicting autism diagnosis

The overall polynomial regression model predicted autism diagnosis (*R* ^*2*^ = 0.52, *p* < 1 × 10^−100^) in males and females (see Table 2 and Figure 1). Sex was associated with autism, such that females had a decreased probability for diagnosis (OR = 0.6 [0.43–0.83, 95% CI], *p* = 0.002). A small sized yet significant association between age and decrease in probability was also found (OR = 0.99 [0.986–0.998, 95% CI], *p* = 0.02).

**TABLE 2:** Polynomial regression with response surface parameters predicting autism diagnosis

	Males			Females		
Effect	Estimate	*p* value	OR [95% CI]	Estimate	*p* value	OR [95% CI]
CE	−0.87 (0.12)	5 × 10^−13^	0.42*** [0.33–0.52]	−1.42 (0.11)	3 × 10^−37^	0.24*** [0.19–0.3]
EE	0.034 (0.11)	0.77	1.03 [0.82–1.3]	−0.18 (0.09)	0.045	0.84* [0.7–0.99]
CE^2^	0.45 (0.07)	9 × 10^−11^	1.57*** [1.37–1.79]	0.26 (0.065)	0.000065	1.3*** [1.14–1.47]
EE^2^	0.22 (0.06)	0.0007	1.25** [1.09–1.42]	0.15 (0.05)	0.003	1.17** [1.05–1.29]
CE × EE	−0.095 (0.075)	0.2	0.91 [0.78–1.05]	−0.045 (0.07)	0.525	0.95 [0.83–1.1]

Response surface parameters							Group Comparison (*p* value)
a1	−0.84 (0.15)	2 × 10^−8^	0.43*** [0.24–0.675]	−1.6 (0.12)	9 × 10^−39^	0.2*** [0.14–0.28]	0.00008***
a2	0.57 (0.08)	1 × 10^−11^	1.78*** [1.4–2.25]	0.37 (0.076)	1 × 10^−6^	1.45*** [1.17–1.79]	0.073
a3	−0.9 (0.18)	9 × 10^−7^	0.4*** [0.24–0.675]	−1.24 (0.16)	8 × 10^−15^	0.29*** [0.18–0.45]	0.16
a4	0.77 (0.15)	3 × 10^−7^	2.15*** [1.42–3.26]	0.46 (0.14)	0.0008	1.585** [1.08–2.32]	0.13

*Notes*: Autism diagnosis prediction using polynomial regression with response surface analysis and parameters statistics of empathy. The response surface parameters (a1–a4) are shown with a1—the linear effect of total empathy, a2—the curvilinear effect of total empathy, a3—the linear effect of empathic disequilibrium, and a4—the curvilinear effect of empathic disequilibrium. Sex differences are depicted. *p < 0.05; **p < 0.005; ***p < 0.0005.

Abbreviations: CE, cognitive empathy; EE, emotional empathy.

Total empathy—Lower total empathy was associated with an autism diagnosis, showing both a linear (*a*1) and a curvilinear (*a*2) association. The linear effect of total empathy was stronger for females than for males (*t* = −3.95, *p* = 0.00008).

Empathic disequilibrium ‐ empathic disequilibrium also significantly predicted autism. A higher probability of autism diagnosis was found in individuals whose EE was higher than their CE (negative *a*3). A significant curvilinear association showed that autism probability increases more sharply as empathic disequilibrium increases (positive *a*4).

#### Autistic traits prediction

**FIGURE 3:**
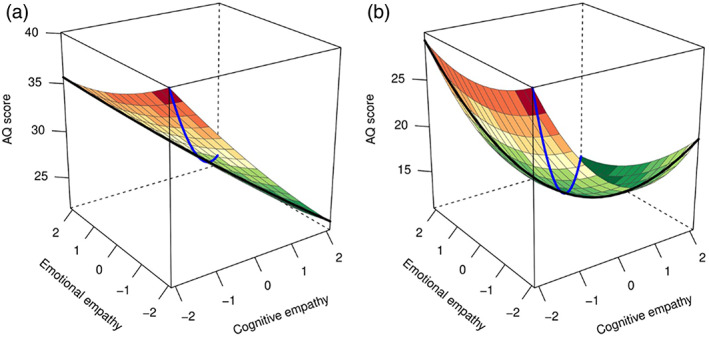
Polynomial regression plot predicting autism‐spectrum quotient (AQ). A plot of the polynomial regression with response surface analysis predicting AQ scores, predicting autism‐quotient score in (a) autistic individuals (*N* = 1905), and (b) non‐autistic individuals (*N* = 3009). The black line represents empathic disequilibrium and the blue line represents total empathy

The overall polynomial regression also predicted autistic traits (*R* ^2^ = 0.75, *p* < 1 × 10^−100^) in the autistic and typical populations (see Table 3 and Figure 3).

**TABLE 3:** Polynomial regression with response surface parameters predicting autism‐spectrum quotient

	Autistic individuals			Non‐autistic individuals		
Effect	Estimate (SE)	*p* value	*β*	Estimate (SE)	*p* value	*β*
CE	−3.62 (0.46)	5 × 10^−15^	−0.265***	−3.15 (0.13)	5 × 10^−115^	−0.19***
EE	−0.5 (0.37)	0.17	−0.03	−1.14 (0.14)	3 × 10^−16^	−0.07***
CE^2^	0.18 (0.22)	0.425	0.027	1.6 (0.13)	5 × 10^−36^	0.125***
EE^2^	0.235 (0.14)	0.1	0.03	0.44 (0.12)	0.0002	0.04***
CE × EE	0.265 (0.22)	0.24	0.03	−0.45 (0.14)	0.0015	−0.03**

Response surface parameters							Group comparison (*p* value)
a1	−4.13 (0.52)	3 × 10^−15^	−0.295***	−4.29 (0.15)	1 × 10^−163^	−0.26***	0.76
a2	0.68 (0.24)	0.036	0.087*	1.59 (0.14)	1 × 10^−28^	0.135***	0.001**
a3	−3.11 (0.65)	2 × 10^−6^	−0.235***	−2 (0.14)	3 × 10^−18^	−0.12***	0.11
a4	0.15 (0.44)	0.74	0.027	2.5 (0.29)	8 × 10^−18^	0.195***	0.00001***

*Notes*: Parameters of the polynomial regression with response surface analysis of emotional empathy (EE) and cognitive empathy (CE), predicting Autism‐Spectrum Quotient score in autistic and non‐autistic individuals. The response surface parameters (a1–a4) are shown with a1—the linear effect of total empathy, a2—the curvilinear effect of total empathy, a3—the linear effect of empathic disequilibrium, and a4—the curvilinear effect of empathic disequilibrium. *p < 0.05, **p < 0.005, ***p < 0.0005.

As expected, autism diagnosis was associated with higher autistic traits (*β =* 0.56, *p* < 1 × 10^−100^), and males showed higher autistic traits than females (*β =* 0.04, *p* = 3 × 10^−7^). Age was also associated with autistic traits (*β =* 0.03, *p* = 0.000009).

Total empathy—Lower total empathy was associated with higher AQ scores in autistic and non‐autistic individuals, showing linear (*a*1) and curvilinear (*a*2) associations. The curvilinear association for total empathy was stronger for non‐autistic individuals (*t* = −3.3, *p* = 0.001).

Empathic disequilibrium—A linear association between empathic disequilibrium and autistic traits was found for both autistic and typical individuals, with higher EE‐dominance predicting higher autistic traits (negative a3). A curvilinear association of empathic disequilibrium and autistic traits was also found for typical individuals, which differed from the non‐significant curvilinear effect of empathic disequilibrium in autistic individuals (*t* = −4.43, *p* = 0.00001).

The polynomial regression models for each of the five AQ subscales are reported in the Appendix S1.

#### Systemizing

**FIGURE 4:**
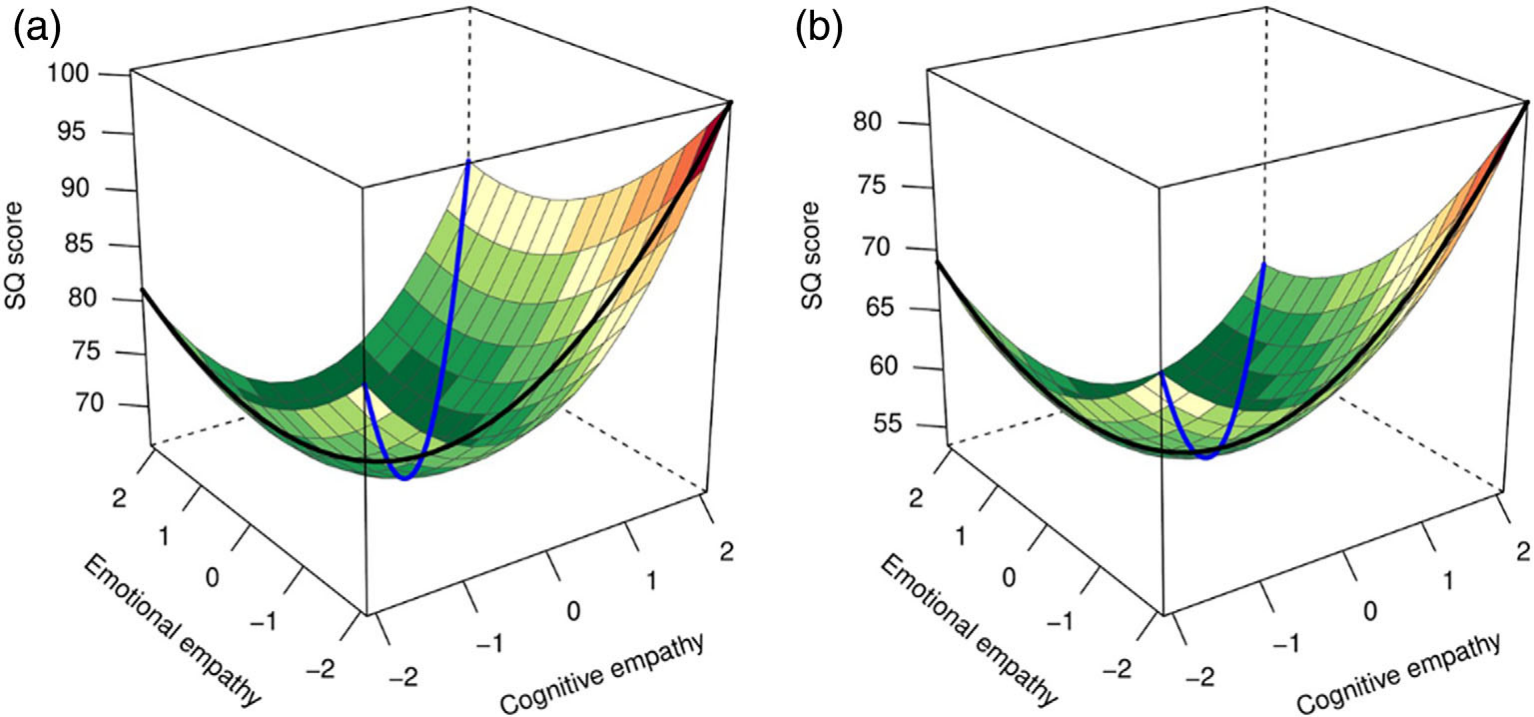
Polynomial regression plot predicting systemizing quotient (SQ). A plot of the polynomial regression with response surface analysis predicting systemizing‐quotient (SQ) score in (a) autistic individuals (*N* = 1809), and (b) non‐autistic individuals (*N* = 2803). The black line represents empathic disequilibrium and the blue line represents total empathy

Finally, we examined the non‐social domain of autism (Baron‐Cohen, 2006; Warrier et al., 2019). Autism diagnosis was associated with higher SQ score (*β =* 0.235, *p* = 3x10^−14^), and males showed higher SQ scores than females (*β = −*0.14, *p* = 4 × 10^−25^). Age was also associated with systemizing (*β =* 0.056, *p* = 0.00001). The overall model of empathy was found to be predictive of systemizing traits (*R* ^*2*^ = 0.26, *p* < 1 × 10^−100^) in autistic and typical individuals. See details in Table 4 and Figure 4.

**TABLE 4:** Polynomial regression with response surface parameters predicting systemizing quotient

	Autistic individuals			Non‐autistic individuals		
Effect	Estimate (SE)	*p* value	*β*	Estimate (SE)	*p* value	*β*
CE	2.35 (1.69)	0.16	0.09	0.64 (0.48)	0.19	0.02
EE	−2.28 (1.3)	0.0785	−0.08	−2.98 (0.5)	4 × 10^−9^	−0.09***
CE^2^	3.4 (0.81)	0.00003	0.26***	2.45 (0.45)	7 × 10^−8^	0.1***
EE^2^	1.32 (0.51)	0.009	0.09*	1.3 (0.43)	0.0025	0.05**
CE × EE	−0.58 (0.79)	0.47	−0.04	−1.13 (0.51)	0.027	−0.04*

Response surface parameters							Group Comparison (*p* value)
a1	0.07 (1.87)	0.97	0.01	−2.35 (0.54)	0.00002	−0.07***	0.21
a2	4.14 (0.84)	9 × 10^−7^	0.31***	2.61 (0.51)	3 × 10^−7^	0.11***	0.12
a3	4.63 (2.365)	0.05	0.17	3.62 (0.82)	0.00001	0.11***	0.685
a4	5.29 (1.57)	0.0008	0.39**	4.87 (1.03)	2 × 10^−6^	0.19***	0.82

*Note*: Parameters of polynomial regression with response surface analysis of emotional empathy (EE) and cognitive empathy (CE), predicting Systemizing‐Quotient score in autistic and non‐autistic individuals. The response surface parameters (a1‐a4) are shown with a1—the linear effect of total empathy, a2—the curvilinear effect of total empathy, a3—the linear effect of empathic disequilibrium, and a4—the curvilinear effect of empathic disequilibrium. *p < 0.05, **p < 0.005, ***p < 0.0005.

Total empathy—In the autistic population, the curvilinear, but not linear, association was significant, with higher SQ scores predicted by high or low total empathy. In contrast, although not significantly different from the autistic group (see ‘group ‘comparison’ statistics in Table 4), typical individuals also showed significant linear and curvilinear association of small sized effects between total empathy and systemizing.

Empathic disequilibrium—In the autistic group, the curvilinear association was again significant, with higher systemizing predicted by empathic disequilibrium. Although only nominally significant, linear association between empathic disequilibrium and SQ showed a tendency towards higher systemizing in autistic individuals with CE‐dominance. In the non‐autistic group, empathic disequilibrium was associated linearly and curvilinearly with empathic disequilibrium, with a tendency towards CE‐dominance predicting systemizing.

## DISCUSSION

In line with our hypotheses, empathic disequilibrium and empathy independently predicted autism diagnosis and autistic traits in autistic and non‐autistic individuals. Specifically, lower total empathy and higher empathic disequilibrium towards EE‐dominance (higher emotional than cognitive empathy) predicted a greater likelihood of autism diagnosis, with additional non‐linear effects which relate to even higher/lower probability at the extremes of empathy and empathic disequilibrium. We also found that EE‐dominance is related to the social domain of autism, while CE‐dominance is related to the non‐social domain. In addition, females across diagnostic groups showed a greater tendency towards higher EE than CE.

This study provides empirical evidence that empathic disequilibrium is at least as informative as empathy for predicting autism diagnosis and is also valuable for predicting autistic traits in both autistic and non‐autistic populations. Empathic disequilibrium offers a new understanding of how autistic people might experience empathy that could solve some of the inconsistencies reported when EE and CE are examined separately (Mazza et al., 2014; Mul et al., 2018). Altogether, such conceptualization might better coincide with the inner experience of some autistic individuals reporting “excess of empathy” (Gillespie‐Lynch et al., 2017) and move beyond the still prevailing conceptualization that autism is associated with a lack of empathy, leading to stigmatization and a negative impact on the autistic community (Fletcher‐Watson & Bird, 2020).

Empathic disequilibrium could advance the field by generating novel hypotheses. For example, we showed that, on average, autistic people tend to show empathic disequilibrium with a tendency towards EE‐dominance, but how would such an imbalance manifest? A person with empathic disequilibrium towards EE‐dominance might understand others' mental states (CE), but her/his ability to respond to these mental states (EE) will be relatively higher. Smith (2009) theoretically suggested that this state could cause overarousal, as the individual becomes overwhelmed with the other's emotions. The cognitive and behavioral characteristics of autistic individuals might constitute an adaptive response to overarousal.

The idea that empathy might be linked to overarousal in autism is also reflected by the non‐linear associations between empathy (both total empathy and empathic disequilibrium), autism diagnosis, and some autistic traits in our study. Some researchers suggest that extreme (high or low) levels of empathy can lead to overarousal and impair psychological functioning (Schipper & Petermann, 2013; Tully et al., 2016). If this is the case in autism – where problems in emotion‐regulation are common (Cai et al., 2018; Mazefsky et al., 2013) ‐ emotional dysregulation may be driven by empathic disequilibrium or extreme levels of total empathy. While it is clear that empathy and emotion regulation are interrelated, this field is in its infancy and the links are currently unclear (Thompson et al., 2019). Further investigation into the mechanism hypothesized here between empathic disequilibrium and problems in emotion‐regulation could shed light on the role of emotion‐regulation in empathy, and the possible involvement of such a link in the manifestation of some autistic traits.

Our findings also suggest that not only is empathic disequilibrium towards EE‐dominance important (since it is associated with social autistic traits), but also empathic disequilibrium towards CE‐dominance, because it is associated with non‐social autistic traits. This was particularly strong in the non‐autistic population, fully replicating our previous results in a neurotypical population (Shalev & Uzefovsky, 2020). Thus, while most autistic individuals lean towards EE‐dominance, CE‐dominance is also associated, albeit to a lesser extent, with an autism diagnosis. This highlights the possible involvement of CE‐dominance in autism as well. It is possible that both extremes of empathic disequilibrium are associated with autism through different pathways – EE‐dominance through difficulties in the social domain and CE‐dominance through the non‐social domain. This interpretation is consistent with a rich literature supporting deviation from homeostasis in either direction underlying the autism spectrum the autistic spectrum (e.g., Auerbach et al., 2011; Jeste & Geschwind, 2014; Tatavarty et al., 2020; Villa et al., 2022). This suggests that the concept of empathic disequilibrium may help us focus on one of the sources of this manifest heterogeneity based on individuals' distinct empathic disequilibrium profiles.

The variability characterizing autism is also reflected in sex differences (Bedford et al., 2020). Studies suggest that autistic females tend to show different characteristics than autistic males, such as increased use of masking of their social difficulties (Hull et al., 2020). Together with the relatively limited research on autistic females, high rates of misdiagnosis have been suspected, stressing the need to better define this group (Kreiser & White, 2014; Loomes et al., 2017). Previous research examining sex differences in empathy could not differentiate autistic males and females based on self‐reported empathy measured by the EQ (for a systematic review, see Kok et al., 2016). However, here we observed average sex differences in empathic disequilibrium, with autistic females displaying EE‐dominance more strongly than autistic males. This suggests that empathic disequilibrium might be of particular relevance to autistic females, providing a new lead into the emotional and cognitive characteristics of autistic females that should be further pursued.

Although substantial differences in empathic disequilibrium were found between autistic and non‐autistic individuals, our data suggest that empathic disequilibrium does not uniquely characterize autism and could be informative for the non‐autistic population as well, where it is associated with autistic traits. Therefore, empathic disequilibrium could provide new avenues of research that are not limited to the autistic population. For instance, empathic disequilibrium could be utilized to generate new hypotheses regarding the development of empathy. Throughout early development, EE remains relatively stable while CE steadily increases (Uzefovsky & Knafo‐Noam, 2017). This might suggest that children show age‐typical EE‐dominant responses, and these diminish throughout early development until reaching balance in adulthood. Although our sample has a relatively large age range, it included only adults. Therefore, we were unable to examine earlier empathic disequilibrium development. Indeed, extensive changes in brain networks underlying empathic components and the connectivity between them occur during earlier periods of development until maturity in early adulthood (Decety, 2010; Decety & Michalska, 2010; Gogtay et al., 2004). Future research adopting a developmental approach in younger participants could clarify how and when some individuals come to experience empathic disequilibrium in adulthood while others do not.

Our study has several limitations. First, all measures used in our study are self‐report questionnaires. Although these measures are validated and correlate with other behavioral measures (Baron‐Cohen et al., 2001; Baron‐Cohen et al., 2003; Baron‐Cohen & Wheelwright, 2004; Lawrence et al., 2004), they primarily reflect the participants' perception of their own functioning and ability. However, while observational methods offer rich information, empathy is largely an internally experiential process that cannot be inferred from behavior alone (Hoffman, 2001), suggesting that self‐report measures are valuable tools for understanding empathy.

Second, the use of questionnaires filled online further limits our conclusions. Participating in such a study requires interest in research, the ability to sign up for it, and the ability to fill it out, suggesting that the sample used in our study consists mainly of autistic individuals with average or above‐average intelligence, likely excluding individuals with intellectual disabilities. As such, our sample does not represent the entire autistic spectrum, where intellectual disability is common and reflects part of the heterogeneity characterizing autism (Lecavalier et al., 2011). Future research examining empathic disequilibrium using behavioral measures of empathy could overcome these limitations and benefit from extending our findings to the entire autistic spectrum. We see the current work as a proof‐of‐concept for the importance of empathic disequilibrium, and this can serve as the basis for developing and validating a behavioral measure for empathic disequilibrium measurement across the autistic spectrum.

Furthermore, the non‐autistic group also included family members of diagnosed individuals, suggesting that this group might be representative of the broad autism phenotype, i.e., people who carry genetic liability for autism and/or display milder phenotypic features (Hurst et al., 2007; Piven et al., 1997). Yet even in this population as a comparison group, we see significant differences as compared to the autistic group.

This study focuses on empathic disequilibrium as an informative index for the diagnosis of autism and for predicting autistic traits in both autistic and non‐autistic populations. By offering a novel way to examine the role of empathy in autism, empathic disequilibrium may better reflect the experience of some autistic individuals. Further research is needed to translate these insights into tools for prognosis, diagnosis, and intervention in autism.

## CONFLICT OF INTEREST

All of the funding organizations had no role in any part of the study including the design and conduct of the study, collection, analysis, interpretation of data, or the writing of the manuscript. All authors declare no conflicts of interest.

## ETHICS STATEMENT

Ethical approval for the research database was obtained from the Psychology Research Ethics Committee, University of Cambridge, United Kingdoms.

## Supporting information


**Appendix S1:** Supporting Information

## Data Availability

The data that support the findings of this study are available on request from the corresponding author. The data are not publicly available due to privacy or ethical restrictions.
