# Supplementary material for: Reexamining empathy in autism: Empathic disequilibrium as a novel predictor of autism diagnosis and autistic traits
Source: Autism Res. 2022 Aug 20;15(10):1917–28. doi: 10.1002/aur.2794 (PMC9804307; doi:10.1002/aur.2794)
Supplement: Supplementary file 1 — Appendix S1: Supporting Information [file AUR-15-1917-s001.docx]

**Supplementary Information**

**Reexamining empathy in autism: Empathic disequilibrium as a novel predictor of autism diagnosis and autistic traits**

Ido Shalev, Varun Warrier, David M. Greenberg, Paula Smith, Carrie Allison, Simon Baron-Cohen, Alal Eran, Florina Uzefovsky

[Confirmatory factor analysis of the Empathy Quotient (EQ) 2](#_Toc109035325)

[Autism-Spectrum Quotient subscales analyses 3](#_Toc109035326)

[Social skill. 3](#_Toc109035327)

[Communication. 4](#_Toc109035328)

[Imagination. 5](#_Toc109035329)

[Attention switching. 7](#_Toc109035330)

[Attention to detail. 8](#_Toc109035331)

[Difference score analysis of empathic disequilibrium and total empathy 10](#_Toc109035332)

[Main analysis after age exclusion 11](#_Toc109035333)

[Predicting autism diagnosis. 11](#_Toc109035334)

[Predicting autistic traits. 11](#_Toc109035335)

[Predicting systemizing. 12](#_Toc109035336)

Confirmatory factor analysis of the Empathy Quotient (EQ)

Two three-factor structures are commonly used in the EQ to tap cognitive, emotional, and social skills aspects of empathy.[^2^](#_ENREF_2)^,^[^3^](#_ENREF_3) To decide which of the two classifications provides the best fit for the specific data used, we conducted a confirmatory factor analysis using lavaan package in R.[^4^](#_ENREF_4) This analysis revealed a reasonable fit for both Lawrence's 28-items three factors and Muncer and Ling's 15-items three factors, but as the latter showed better fit indices (see Table S1), we chose to calculate emotional empathy (EE) and cognitive empathy (CE) scores using Muncer and Ling's classification.[^2^](#_ENREF_2) We did not include the subscale tapping social skills as it does not directly relate to EE and CE.

**Table S1.** Confirmatory factor analysis and model fit parameters of Lawrence and Shaw, and Muncer and Ling Empathy Quotient classification

| **Model** | **Items** | **Goodness of fit indices** | | | | | | |
| --- | --- | --- | --- | --- | --- | --- | --- | --- |
|  |  | **X^2^** | **df** | **X^2^/df** | **RMSEA** | **-2 Log Likelihood** | **AIC** | **BIC** |
| **Lawrence and Shaw** | 28 | 9097 | 347 | 26.22 | 0.07 | 266,712 | 266,830 | 267,216 |
| **Muncer and Ling** | 15 | 1773 | 87 | 20.38 | 0.062 | 149,985 | 150,051 | 150,267 |

Autism-Spectrum Quotient subscales analyses

To understand further the implications of empathic disequilibrium on the autism-related measures described in the main text, we conducted exploratory analyses of the subscales of the Autism-Spectrum Quotient (AQ).[^1^](#_ENREF_1) AQ comprised of five different subscales: ‘social skill’, ‘communication’, ‘imagination’, ‘attention switching’, and ‘attention to detail’.

In the below analyses, we followed the same methodology described in the main article. Meaning, total empathy and empathic disequilibrium were measured using Empathy Quotient (EQ).[^2^](#_ENREF_2) Polynomial regression analysis[^3^](#_ENREF_3)^,^[^4^](#_ENREF_4) was conducted to simultaneously investigate the association between empathic disequilibrium, total empathy and autistic traits in each subscale. Differences in surface parameters were investigated between autistic and non-autistic individuals. Age and sex were used as covariates. As each of the five subscales of AQ was examined separately, we used a strict Bonferroni correction of p < 0.05/5 subscales = 0.01 to account for multiple testing.

Social skill.

The overall polynomial regression of empathy predicted autistic traits measured using the AQ (*R^2^* = 0.65, *p* < 1x10^-100^) in autistic and non-autistic populations (see Table S1 and Figure S1). Autism diagnosis was associated with higher autistic traits in the social skill domain (*β* = 0.52, *p* < 1x10^-100^), and males showed higher autistic traits in this domain than females (*β* = 0.06, *p* = 9x10^-11^). Age was also associated with autistic traits in this subscale (*β* = 0.08, *p* = 1x10^-21^).

**Table S2. Polynomial regression with response surface parameters predicting the ‘social skill’ subscale.**

|  | **Autistic individuals** | | | **non-autistic individuals** | | |  |
| --- | --- | --- | --- | --- | --- | --- | --- |
| **Effect** | **Estimate (SE)** | **p-value** | **beta** | **Estimate (SE)** | **p-value** | **beta** |  |
| **CE** | -0.95 (0.15) | 2x10^-10^ | -0.26*** | -1.08 (0.04) | 2x10^-130^ | -0.24*** |  |
| **EE** | -0.225 (0.12) | 0.06 | -0.055 | -0.36 (0.04) | 6x10^-16^ | -0.08*** |  |
| **CE^2^** | -0.03 (0.07) | 0.695 | -0.015 | 0.52 (0.04) | 7x10^-37^ | 0.15*** |  |
| **EE^2^** | 0.004 (0.05) | 0.92 | 0.002 | 0.12 (0.04) | 0.0016 | 0.04* |  |
| **CE x EE** | 0.11 (0.07) | 0.12 | 0.05 | -0.17 (0.05) | 0.00025 | -0.04** |  |
| **Response surface parameters** | | | | | | | **Group Comparison** |
| **a1** | -1.17 (0.17) | 2x10^-12^ | -0.31*** | -1.4 (0.05) | 2x10^-178^ | -0.33*** | 0.62 |
| **a2** | 0.09 (0.075) | 0.87 | 0.04 | 0.47 (0.05) | 6x10^-25^ | 0.145*** | 0.00001*** |
| **a3** | -0.72 (0.21) | 0.0055 | -0.2* | -0.72 (0.07) | 2x10^-22^ | -0.16*** | 1 |
| **a4** | -0.135 (0.14) | 0.95 | -0.07 | 0.81 (0.09) | 3x10^-18^ | 0.23*** | 3x10^-8^*** |

**p* < 0.01, ***p* < 0.001, ****p* < 0.0001

Parameters of the polynomial regression with response surface analysis of emotional empathy (EE) and cognitive empathy (CE), predicting ‘social skill’ subscale of the Autism-Spectrum Quotient score in autistic and non-autistic individuals. The response surface parameters (a1-a4) are shown with a1 representing the linear effect of total empathy, a2 - the curvilinear effect of total empathy, a3 - the linear effect of empathic disequilibrium, and a4 – the curvilinear effect of empathic disequilibrium.

**Fig. S1. Polynomial regression plot predicting the ‘social skill’ subscale.** A Plot of the polynomial regression with response surface analysis predicting Autism-Spectrum Quotient (AQ) score of the ‘social skill’ subscale, predicting autism-quotient score in a. autistic individuals (N = 1,905), and b. non-autistic individuals (N = 3,009). The black line represents empathic disequilibrium, and the blue line represents total empathy.


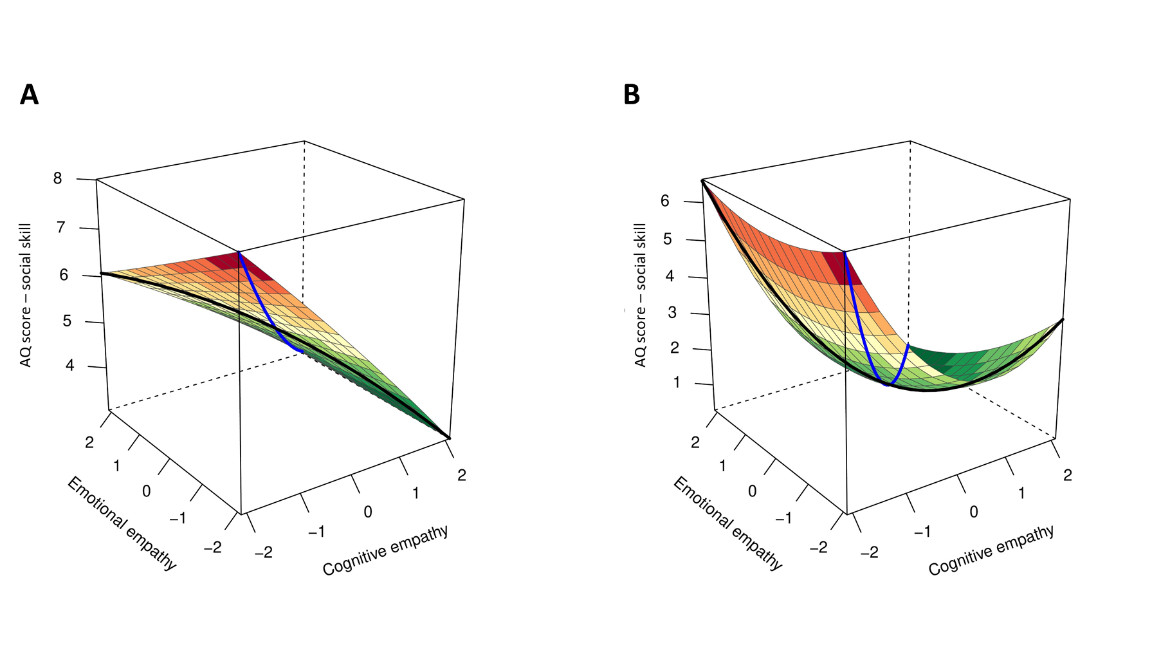


Total empathy - Lower total empathy was associated with higher deficits in social skills in both autistic and non-autistic individuals, showing linear (a1). Curvilinear (a2) association between total empathy and the social skill subscale was found only for non-autistic individuals but not for autistic individuals.

Empathic disequilibrium - A linear association between empathic disequilibrium and autistic traits was found for both autistic and non-autistic individuals, with higher EE-dominance predicting higher autistic traits (negative a3). A curvilinear association of empathic disequilibrium and autistic traits was also found for non-autistic individuals, which differed from the non-significant curvilinear effect of empathic disequilibrium in autistic individuals.

Communication.

The overall polynomial regression of empathy predicted autistic traits measured using the AQ (*R^2^* = 0.72, *p* < 1x10^-100^) in the autistic and the non-autistic population (see Table S2 and Figure S2). Autism diagnosis was associated with higher autistic traits in the communication domain (*β* = 0.54, *p* < 1x10^-100^), and males showed higher autistic traits in this domain than females (*β* = 0.03, *p* = 0.0001). Age was also associated with AQ scores of the ‘communication’ subscale (*β* = 0.025, *p* = 0.0009).

**Table S3. Polynomial regression with response surface parameters predicting the ‘communication’ subscale.**

|  | **Autistic individuals** | | | **non-autistic individuals** | | |  |
| --- | --- | --- | --- | --- | --- | --- | --- |
| **Effect** | **Estimate (SE)** | **p-value** | **beta** | **Estimate (SE)** | **p-value** | **beta** |  |
| **CE** | -1 (0.13) | 2x10^-14^ | -0.28*** | -0.92 (0.04) | 1x10^-122^ | -0.21*** |  |
| **EE** | 0.02 (0.1) | 0.85 | 0.005 | -0.23 (0.04) | 7x10^-9^ | -0.05*** |  |
| **CE^2^** | 0.06 (0.06) | 0.3 | 0.04 | 0.48 (0.04) | 2x10^-40^ | 0.14*** |  |
| **EE^2^** | 0.12 (0.03) | 0.0002 | 0.05** | 0.12 (0.03) | 0.0002 | 0.04** |  |
| **CE x EE** | 0.05 (0.06) | 0.43 | 0.02 | -0.16 (0.04) | 0.00009 | -0.04*** |  |
| **Response surface parameters** | | | | | | | **Group Comparison** |
| **a1** | -0.98 (0.15) | 3x10^-11^ | -0.27*** | -1.14 (0.04) | 3x10^-148^ | -0.26*** | 0.27 |
| **a2** | 0.21 (0.07) | 0.0017 | 0.11* | 0.44 (0.04) | 2x10^-28^ | 0.14*** | 0.002* |
| **a3** | -1.02 (0.18) | 3x10^-8^ | -0.28*** | -0.69 (0.06) | 2x10^-26^ | -0.16*** | 0.09 |
| **a4** | 0.11 (0.12) | 0.38 | 0.06 | 0.76 (0.08) | 1x10^-20^ | 0.22*** | 0.00001*** |

Parameters of polynomial regression with response surface analysis of emotional empathy (EE) and cognitive empathy (CE), predicting ‘communication’ subscale of the Autism-Spectrum Quotient in autistic and non-autistic individuals. The response surface parameters (a1-a4) are shown with a1 representing the linear effect of total empathy, a2 - the curvilinear effect of total empathy, a3 - the linear effect of empathic disequilibrium, and a4 – the curvilinear effect of empathic disequilibrium.

**p* < 0.01, ***p* < 0.001, ****p* < 0.0001


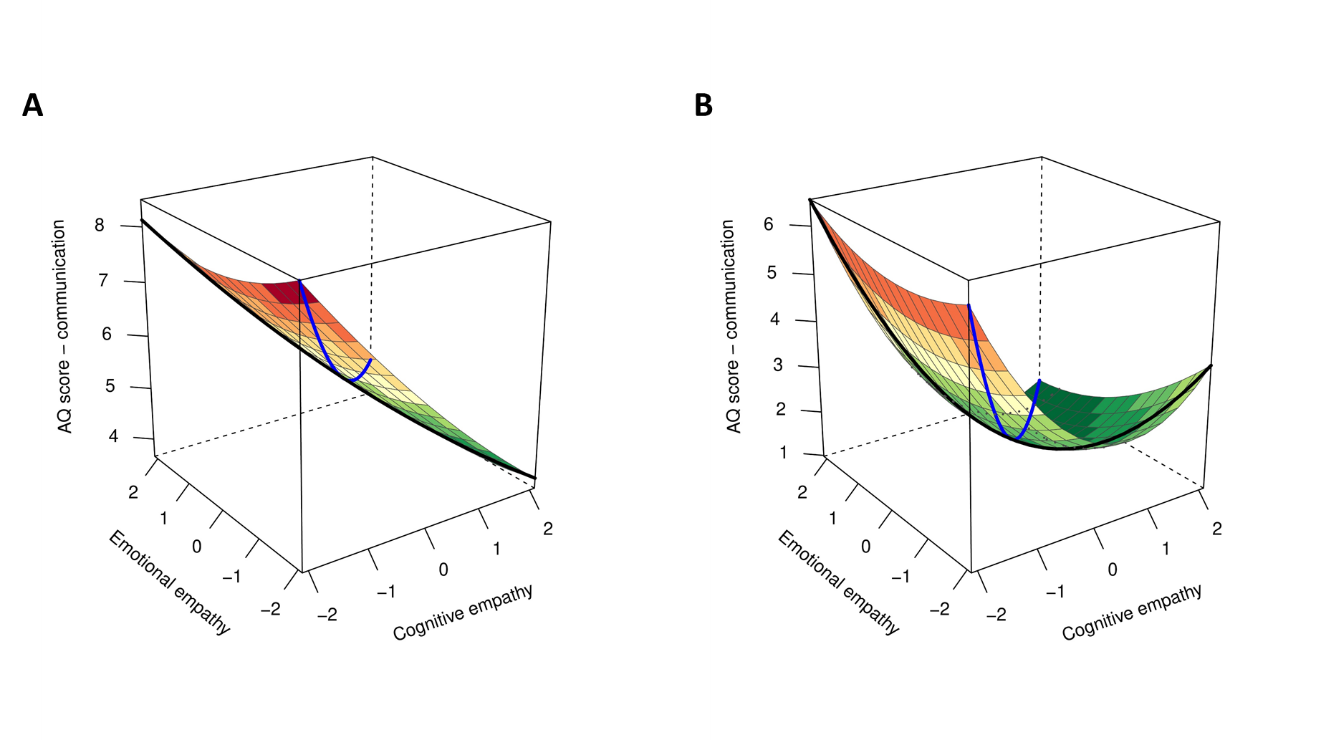


**Fig. S2. Polynomial regression plot predicting the ‘communication’ subscale.** A Plot of the polynomial regression with response surface analysis predicting Autism-Spectrum Quotient (AQ) score of the ‘communication’ subscale, predicting autism-quotient score in a. autistic individuals (N = 1,905), and b. non-autistic individuals (N = 3,009). The black line represents empathic disequilibrium, and the blue line represents total empathy.

Total empathy - Lower total empathy was associated with higher deficits in communication in both autistic and non-autistic individuals, showing linear (a1) and curvilinear (a2) associations.

Empathic disequilibrium - A linear association between empathic disequilibrium and autistic traits was found for both autistic and non-autistic individuals, with higher EE-dominance predicting higher autistic traits (negative a3). A curvilinear association of empathic disequilibrium and autistic traits was also found only for non-autistic individuals.

Imagination.

The overall polynomial regression of empathy predicted autistic traits measured using the AQ (*R^2^* = 0.55, *p* < 1x10^-100^) in autistic and non-autistic population (see Table S3 and Figure S3). Autism diagnosis was associated with higher autistic traits in the imagination domain (*β* = 0.38, *p* = 1x10^-59^). Age was associated with the ‘imagination’ subscale (*β* = 0.06, *p* = 2x10^-10^). No sex differences were found (*β* = 0.007, *p* = 0.48).

**Table S4. Polynomial regression with response surface parameters predicting the ‘imagination’ subscale.**

|  | **Autistic individuals** | | | **non-autistic individuals** | | |  |
| --- | --- | --- | --- | --- | --- | --- | --- |
| **Effect** | **Estimate (SE)** | **p-value** | **beta** | **Estimate (SE)** | **p-value** | **beta** |  |
| **CE** | -0.49 (0.14) | 0.0003 | -0.16** | -0.48 (0.04) | 5x10^-33^ | -0.13*** |  |
| **EE** | -0.31 (0.11) | 0.004 | -0.09* | -0.25 (0.04) | 2x10^-9^ | -0.07*** |  |
| **CE^2^** | 0.17 (0.065) | 0.01 | 0.115* | 0.16 (0.04) | 0.00001 | 0.06*** |  |
| **EE^2^** | 0.06 (0.04) | 0.18 | 0.03 | 0.04 (0.03) | 0.31 | 0.01 |  |
| **CE x EE** | 0.04 (0.07) | 0.525 | 0.02 | -0.02 (0.04) | 0.69 | -0.005 |  |
| **Response surface parameters** | | | | | | | **Group Comparison** |
| **a1** | -0.81 (0.15) | 0.0000002 | -0.26*** | -0.73 (0.04) | 1x10^-57^ | -0.2*** | 0.63 |
| **a2** | 0.27 (0.07) | 0.0001 | 0.17*** | 0.18 (0.04) | 0.00001 | 0.06*** | 0.29 |
| **a3** | -0.18 (0.19) | 0.36 | -0.07 | 0.23 (0.07) | 0.0006 | -0.06** | 0.79 |
| **a4** | 0.185 (0.13) | 0.16 | 0.125 | 0.22 (0.09) | 0.01 | 0.08* | 0.84 |

Parameters of polynomial regression with response surface analysis of emotional empathy (EE) and cognitive empathy (CE), predicting ‘imagination’ subscale of the Autism-Spectrum Quotient in autistic individuals and non-autistic individuals. The response surface parameters (a1-a4) are shown with a1 representing the linear effect of total empathy, a2 - the curvilinear effect of total empathy, a3 - the linear effect of empathic disequilibrium, and a4 – the curvilinear effect of empathic disequilibrium.

**p* < 0.01, ***p* < 0.001, ****p* < 0.0001


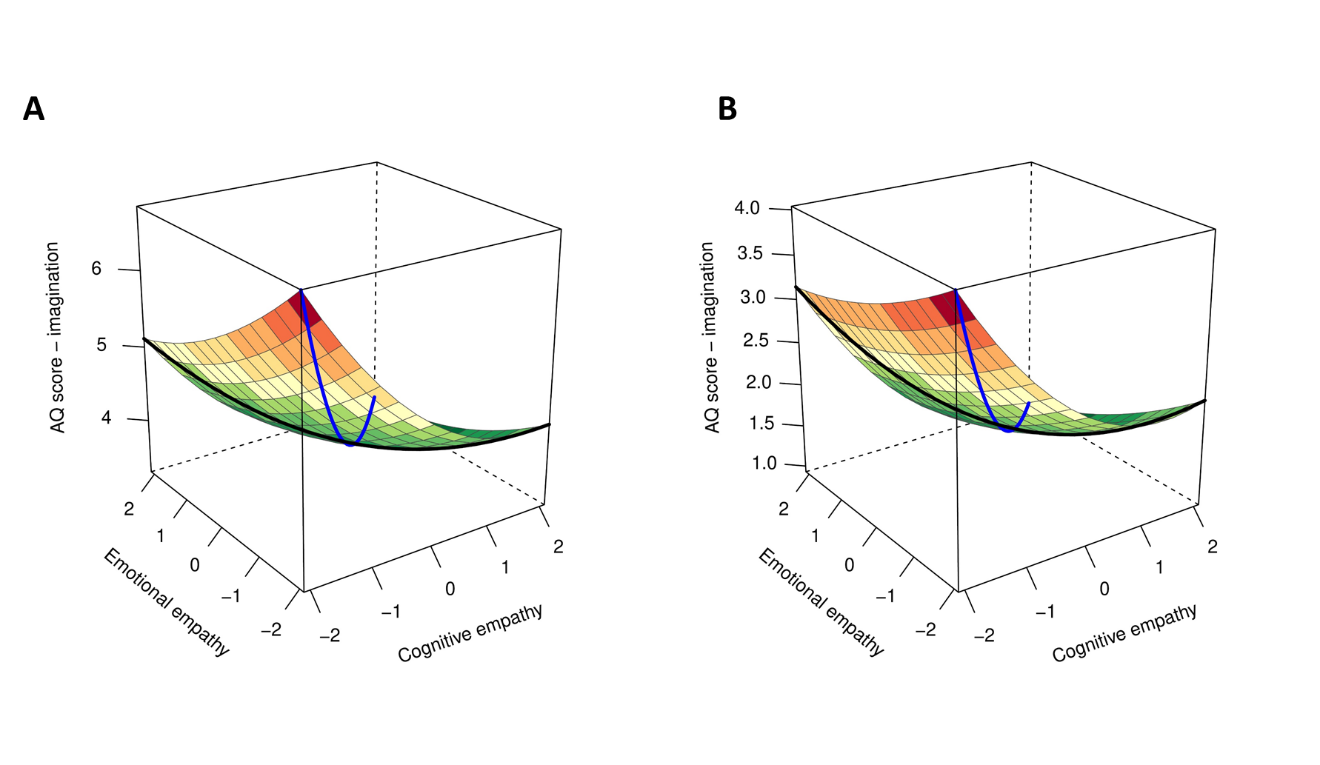


**Fig. S3.** **Polynomial regression plot predicting the ‘imagination’ subscale.** Plot of the polynomial regression with response surface analysis predicting Autism-Spectrum Quotient (AQ) score of the ‘imagination’ subscale, predicting autism-quotient score in a. autistic individuals (N = 1,905), and b. non-autistic individuals (N = 3,009). The black line represents empathic disequilibrium, and the blue line represents total empathy.

Total empathy - Lower total empathy was associated with higher deficits in imagination in both autistic and non-autistic individuals, showing linear (a1) and curvilinear (a2) associations.

Empathic disequilibrium - Linear and curvilinear associations between empathic disequilibrium and autistic traits were found for non-autistic individuals, with higher EE-dominance predicting higher autistic traits (negative a3). Empathic disequilibrium was not associated with deficits in imagination in autistic individuals.

Attention switching.

The overall polynomial regression of empathy predicted autistic traits measured using the AQ (*R^2^* = 0.57, *p* < 1x10^-100^) in autistic and non-autistic populations (see Table S4 and Figure S4). Autism diagnosis was associated with higher autistic traits in the attention switching domain (*β* = 0.54, *p* < 1x10^-100^). Males showed more deficits in attention switching than females (*β* = 0.04, *p* = 0.00005). Age was negatively correlated with deficits in attention switching in this subscale (*β* = -0.03, *p* = 0.0004).

**Table S5. Polynomial regression with response surface parameters predicting the ‘attention switching’ subscale.**

|  | **Autistic individuals** | | | **Non-autistic individuals** | | |  |
| --- | --- | --- | --- | --- | --- | --- | --- |
| **Effect** | **Estimate (SE)** | **p-value** | **beta** | **Estimate (SE)** | **p-value** | **beta** |  |
| **CE** | -0.75 (0.14) | 0.0000001 | -0.23*** | -0.74 (0.04) | 3x10^-69^ | -0.2*** |  |
| **EE** | 0.04 (0.11) | 0.74 | 0.01 | -0.06 (0.04) | 0.155 | -0.02 |  |
| **CE^2^** | -0.005 (0.07) | 0.94 | -0.003 | 0.29 (0.04) | 8x10^-14^ | 0.1*** |  |
| **EE^2^** | 0.02 (0.04) | 0.59 | 0.01 | 0.08 (0.04) | 0.03 | 0.03 |  |
| **CE x EE** | 0.05 (0.07) | 0.45 | 0.03 | -0.08 (0.04) | 0.06 | -0.02 |  |
| **Response surface parameters** | | | | | | | **Group Comparison** |
| **a1** | -0.71 (0.16) | 0.000009 | -0.22*** | -0.8 (0.05) | 1x10^-100^ | -0.21*** | 0.6 |
| **a2** | 0.07 (0.07) | 0.32 | 0.04 | 0.29 (0.04) | 4x10^-11^ | 0.1*** | 0.01* |
| **a3** | -0.78 (0.2) | 0.00009 | -0.245*** | -0.68 (0.07) | 1x10^-100^ | -0.18*** | 0.61 |
| **a4** | -0.03 (0.14) | 0.8 | -0.02 | 0.45 (0.09) | 0.0000004 | 0.15*** | 0.003* |

Parameters of polynomial regression with response surface analysis of emotional empathy (EE) and cognitive empathy (CE), predicting ‘attention switching’ subscale of the Autism-Spectrum Quotient in autistic and non-autistic individuals. The response surface parameters (a1-a4) are shown with a1 representing the linear effect of total empathy, a2 - the curvilinear effect of total empathy, a3 - the linear effect of empathic disequilibrium, and a4 – the curvilinear effect of empathic disequilibrium.

**p* < 0.01, ***p* < 0.001, ****p* < 0.0001


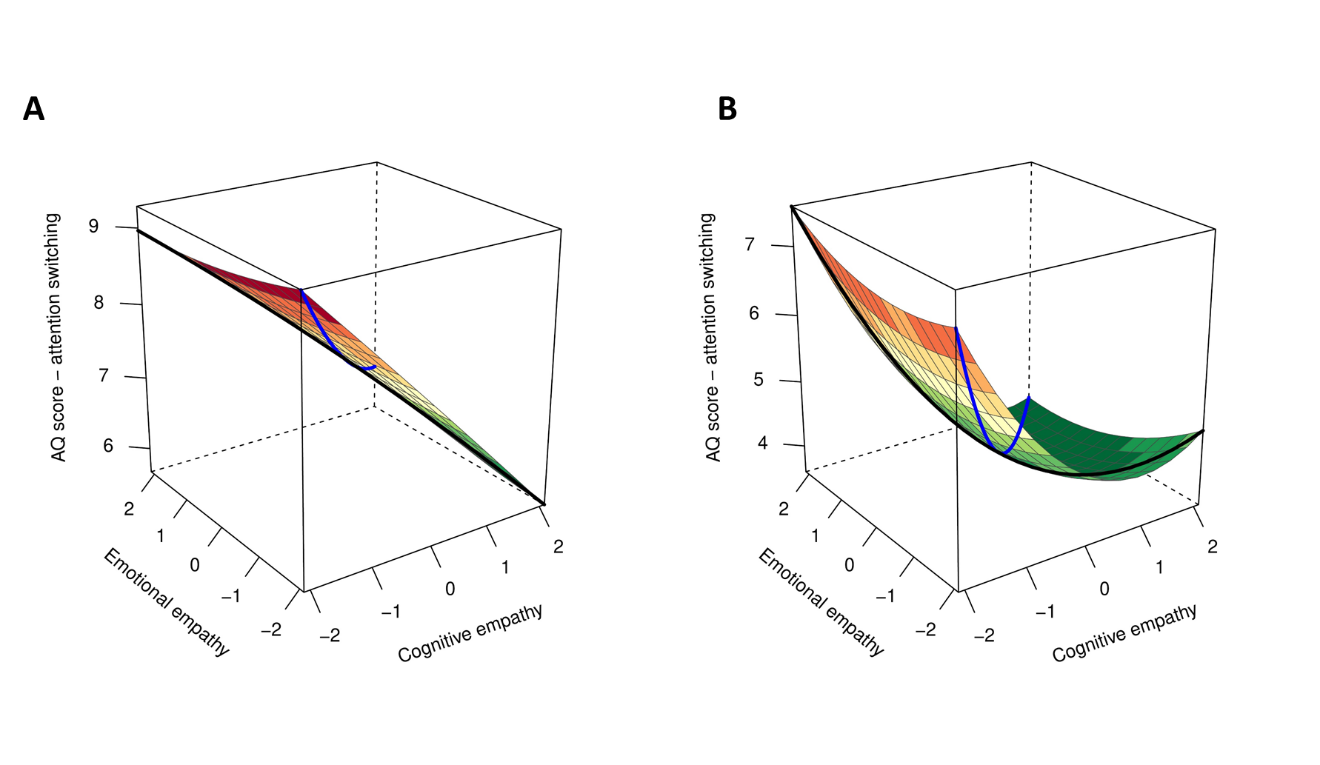


**Fig. S4.** **Polynomial regression plot predicting the ‘attention switching’ subscale.** Plot of the polynomial regression with response surface analysis predicting Autism-Spectrum Quotient (AQ) score of the ‘attention switching’ subscale, predicting autism-quotient score in a. autistic individuals (N = 1,905), and b. non-autistic individuals (N = 3,009). The black line represents empathic disequilibrium, and the blue line represents total empathy.

Total empathy - Lower total empathy was linearly associated with higher deficits in attention switching in both autistic and non-autistic individuals. A curvilinear association between total empathy and deficits in attention switching was found for non-autistic individuals only.

Empathic disequilibrium - A linear association between empathic disequilibrium and autistic traits was found for both autistic and non-autistic individuals, with higher EE-dominance predicting higher autistic traits (negative a3). A curvilinear association of empathic disequilibrium and autistic traits was also found only for non-autistic individuals.

Attention to detail.

The overall polynomial regression of empathy predicted autistic traits measured using the AQ (*R^2^* = 0.21, *p* < 1x10^-100^) in autistic and non-autistic population (see Table S5 and Figure S5). Autism diagnosis was associated with higher autistic traits in the attention to detail domain (*β* = 0.32, *p* = 2x10^-25^). Neither sex (*β* = 0.02, *p* = 0.14) nor age (*β* = -0.015, *p* = 0.25) were associated with the ‘attention to detail’ subscale of the AQ.

**Table S6.** **Polynomial regression with response surface parameters predicting the ‘attention to detail’ subscale.**

|  | **Autistic individuals** | | | **Non-autistic individuals** | | |  |
| --- | --- | --- | --- | --- | --- | --- | --- |
| **Effect** | **Estimate (SE)** | **p-value** | **beta** | **Estimate (SE)** | **p-value** | **beta** |  |
| **CE** | -0.37 (0.16) | 0.017 | -0.14 | 0.07 (0.05) | 0.12 | 0.02 |  |
| **EE** | -0.01 (0.125) | 0.92 | -0.004 | -0.24 (0.05) | 0.0000003 | -0.08*** |  |
| **CE^2^** | -0.005 (0.075) | 0.95 | -0.004 | 0.14 (0.04) | 0.0007 | 0.06** |  |
| **EE^2^** | 0.05 (0.05) | 0.27 | 0.04 | 0.08 (0.04) | 0.05 | 0.03 |  |
| **CE x EE** | 0.015 (0.08) | 0.84 | 0.01 | -0.03 (0.05) | 0.54 | -0.01 |  |
| **Response surface parameters** | | | | | | | **Group Comparison** |
| **a1** | -0.385 (0.18) | 0.03 | -0.15 | -0.17 (0.05) | 0.0008 | -0.055** | 0.25 |
| **a2** | 0.06 (0.08) | 0.42 | 0.04 | 0.2 (0.05) | 0.00005 | 0.08*** | 0.16 |
| **a3** | -0.36 (0.22) | 0.1 | -0.14 | 0.31 (0.08) | 0.00005 | 0.1*** | 0.004* |
| **a4** | 0.03 (0.15) | 0.83 | 0.02 | 0.26 (0.98) | 0.009 | 0.1* | 0.22 |

Parameters of polynomial regression with response surface analysis of emotional empathy (EE) and cognitive empathy (CE), predicting ‘attention to detail’ subscale of the Autism-Spectrum Quotient in autistic and non-autistic individuals. The response surface parameters (a1-a4) are shown with a1 representing the linear effect of total empathy, a2 - the curvilinear effect of total empathy, a3 - the linear effect of empathic disequilibrium, and a4 – the curvilinear effect of empathic disequilibrium.

**p* < 0.01, ***p* < 0.001, ****p* < 0.0001


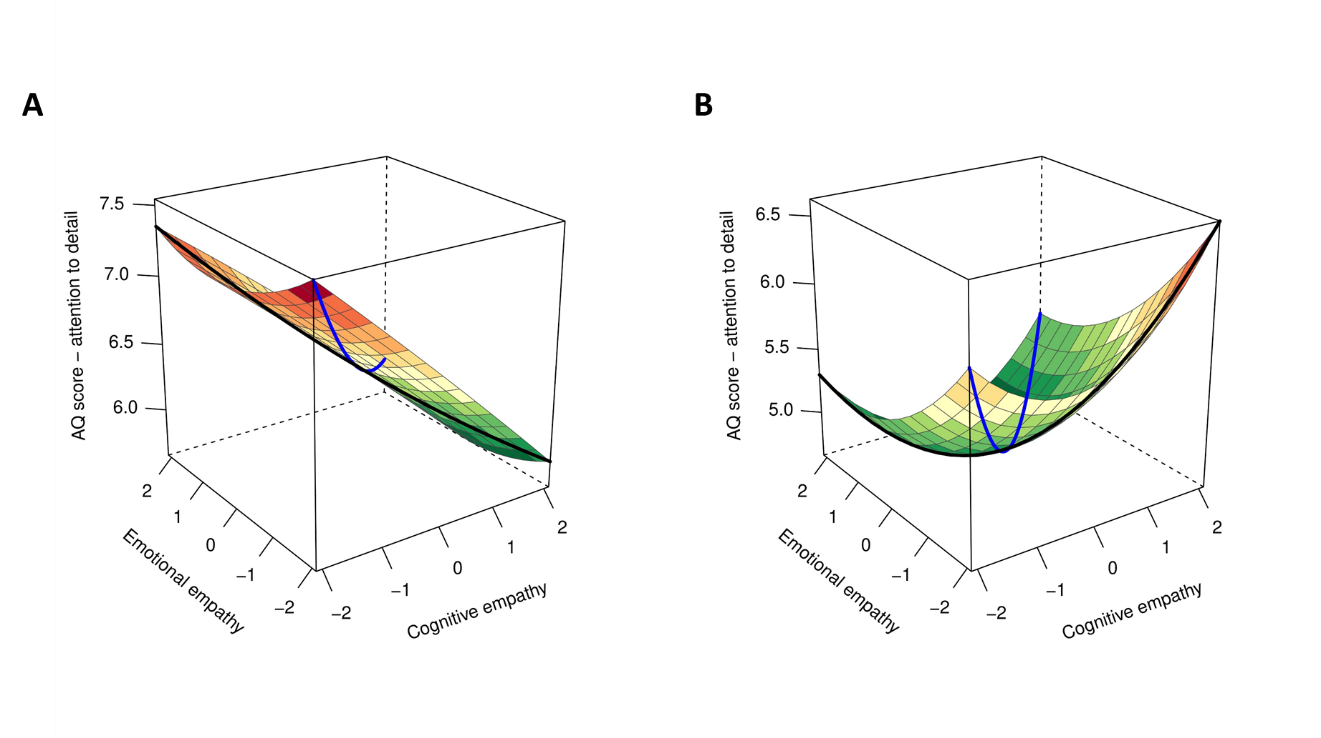


**Fig. S5.** **Polynomial regression plot predicting the ‘attention to detail’ subscale.** Plot of the polynomial regression with response surface analysis predicting Autism-Spectrum Quotient (AQ) score of the ‘attention to detail’ subscale, predicting autism-quotient score in a. autistic individuals (N = 1,905), and b. non-autistic individuals (N = 3,009). The black line represents empathic disequilibrium, and the blue line represents total empathy.

Total empathy - Lower total empathy was associated with higher deficits in attention to detail only in non-autistic individuals, showing linear (a1) and curvilinear (a2) associations. No association between total empathy and deficits in attention to detail was found in autistic individuals.

Empathic disequilibrium - A linear and curvilinear associations between empathic disequilibrium and deficits in attention to detail were found for non-autistic individuals only, with higher CE-dominance predicting higher autistic traits (positive a3). No associations were found between empathic disequilibrium and attention to detail in autistic individuals.

Difference score analysis of empathic disequilibrium and total empathy

To test for the unique contribution of empathic disequilibrium beyond empathy, we calculated empathic disequilibrium by subtracting standardized CE from standardized EE. Multiple regression with both empathic disequilibrium and total empathy within the same model were conducted to predict autism diagnosis (with logistic regression) and traits (using AQ and SQ). The three- and two-way interactions between empathic disequilibrium, sex, and diagnosis were also examined. Age was controlled for as a covariate.

*Autism diagnosis.* empathic disequilibrium and total empathy scores each uniquely predicted autism diagnosis, with empathic disequilibrium being stronger predictor of autism diagnosis than total empathy scores (*b* = -0.14 [95% CI = -0.15, -0.13], OR = 0.87 [95% CI = 0.86, 0.88], *p* < 1x10^-100^ for total empathy; *b* = -0.47 [95% CI = -0.55, -0.38], OR = 0.63 [95% CI = 0.58, 0.68], *p* < 1x10^-100^ for empathic disequilibrium). No interaction was found between empathic disequilibrium and sex (*b* = 0.06 [95% CI = -0.11, 0.23], OR = 1.05, [95% CI = 0.89, 1.26], *p* = 0.51).

*Autistic traits.* EQ score was found to be a strong predictor of autistic traits (*β* = -0.82 [95% CI = -0.83, -0.80], *p* < 1x10^-100^), yet beyond this correlation, empathic disequilibrium still had a unique contribution for the prediction of autistic traits (*β* = -0.09 [95% CI = -0.11, -0.08], *p* = 2x10^-30^). Controlling for total empathy, we also found a three-way interaction between empathic disequilibrium, sex, and diagnosis (*β* = 0.03 [95% CI = 0.01, 0.06], *p* = 0.009). Further analysis revealed a significant simple two-way interaction between males and females in autistic individuals (*β* = 0.06 [95% CI = 0.006, 0.1], *p* = 0.03), showing that empathic disequilibrium was associated with autistic traits only in autistic females (*β* = -0.13 [95% CI = -0.17, -0.07], *p* = 0.01), empathic disequilibrium not in autistic males (*β* = -0.05 [95% CI = -0.11, 0.003], *p* = 0.06). No differences in empathic disequilibrium were found between males and females in the non-autistic group (*p* = 0.051), but a simple effect was significant (*β* = -0.06 [95% CI = -0.08, -0.03], *p* = 0.00006).

*Systemizing.* Beyond total empathy (*β* = -0.47 [95% CI = -0.49, -0.44], *p* < 1x10^-100^), empathic disequilibrium also predicted systemizing (*β* = 0.07 [95% CI = 0.05, 0.1], *p* = 0.00006). No three-way interaction was found between empathic disequilibrium, sex, and diagnosis (*β* = -0.03 [95% CI = -0.07, 0.02], *p* = 0.29). The two-way interactions between empathic disequilibrium and sex (*β* = 0.01 [95% CI = -0.03, 0.06], *p* = 0.56), and empathic disequilibrium and diagnosis (*β* = -0.01 [95% CI = -0.06, 0.03], *p* = 0.63) were also insignificant.

Main analysis after age exclusion

Predicting autism diagnosis.

**Polynomial regression with response surface parameters predicting autism diagnosis after age exclusion.**

|  | **Males** | | | **Females** | | |  |
| --- | --- | --- | --- | --- | --- | --- | --- |
| **Effect** | **Estimate** | **p-value** | **OR [95% CI]** | **Estimate** | **p-value** | **OR [95% CI]** |  |
| **CE** | -0.87 (0.12) | 6x10^-13^ | 0.42***  [0.33 - 0.53] | -1.43 (0.11) | 9x10^-38^ | 0.24 ***  [0.19 - 0.29] |  |
| **EE** | 0.032 (0.12) | 0.78 | 1.03  [0.82 – 1.29] | -0.18 (0.09) | 0.04 | 0.83*  [0.7 - 0.99] |  |
| **CE^2^** | 0.445(0.07) | 1x10^-10^ | 1.56***  [1.36 - 1.78] | 0.26 (0.065) | 0.00005 | 1.3***  [1.14 - 1.48] |  |
| **EE^2^** | 0.22 (0.06) | 0.0006 | 1.25**  [1.10 - 1.42] | 0.16 (0.05) | 0.002 | 1.17**  [1.05 - 1.295] |  |
| **CE x EE** | -0.10 (0.075) | 0.19 | 0.91  [0.78- 1.05] | -0.05 (0.075) | 0.46 | 0.95  [0.83 - 1.09] |  |
| **Response surface parameters** | | | | | | | **Group Comparison (p – value)** |
| **a1** | -0.83 (0.15) | 2x10^-8^ | 0.43***  [0.29 - 0.66] | -1.6 (0.12) | 9x10^-39^ | 0.2***  [0.14 - 0.28] | 0.00006*** |
| **a2** | 0.57 (0.08) | 2x10^-11^ | 1.77***  [1.4 -2.24] | 0.37(0.076) | 1x10^-6^ | 1.45***  [1.17 - 1.79] | 0.08 |
| **a3** | -0.9 (0.18) | 1x10^-6^ | 0.41***  [0.24 - 0.68] | -1.25 (0.16) | 6x10^-15^ | 0.29***  [0.18 - 0.45] | 0.15 |
| **a4** | 0.77 (0.15) | 3x10^-7^ | 2.15***  [1.42 - 3.26] | 0.47(0.14) | 0.0006 | 1.61**  [1.10 - 2.36] | 0.15 |

Autism diagnosis prediction using polynomial regression with response surface analysis and parameters statistics of empathy. The response surface parameters (a1-a4) are shown with a1 representing the linear effect of total empathy, a2 - the curvilinear effect of total empathy, a3 - the linear effect of empathic disequilibrium, and a4 – the curvilinear effect of empathic disequilibrium. Sex differences are depicted. EE – emotional empathy, CE – cognitive empathy.

**p* < 0.05, ***p* < 0.005, ****p* < 0.0005

Predicting autistic traits.

**Polynomial regression with response surface parameters predicting Autism-Spectrum Quotient after age exclusion.**

|  | **Autistic individuals** | | | **Non-autistic individuals** | | |  |
| --- | --- | --- | --- | --- | --- | --- | --- |
| **Effect** | **Estimate (SE)** | **p-value** | **beta** | **Estimate (SE)** | **p-value** | **beta** |  |
| **CE** | -3.64 (0.46) | 5x10^-15^ | -0.27*** | -3.13 (0.13) | 2x10^-113^ | -0.19*** |  |
| **EE** | -0.51 (0.37) | 0.17 | -0.03 | -1.12 (0.14) | 1x10^-15^ | -0.07*** |  |
| **CE^2^** | 0.16 (0.22) | 0.46 | 0.025 | 1.63 (0.13) | 5x10^-37^ | 0.13*** |  |
| **EE^2^** | 0.23 (0.14) | 0.11 | 0.03 | 0.41 (0.12) | 0.0006 | 0.03** |  |
| **CE x EE** | 0.28 (0.23) | 0.22 | 0.04 | -0.44 (0.14) | 0.002 | -0.03** |  |
| **Response surface parameters** | | | | | | | **Group Comparison (p -value)** |
| **a1** | -4.15 (0.52) | 2x10^-15^ | -0.3*** | -4.26 (0.15) | 7x10^-161^ | -0.26*** | 0.84 |
| **a2** | 0.67 (0.24) | 0.04 | 0.092* | 1.60 (0.14) | 9x10^-29^ | 0.13*** | 0.0008** |
| **a3** | -3.13 (0.65) | 2x10^-6^ | -0.23*** | -2 (0.23) | 3x10^-18^ | -0.12*** | 0.10 |
| **a4** | 0.11 (0.445) | 0.99 | 0.030 | 2.49 (0.29) | 1x10^-17^ | 0.19*** | 0.000008*** |

Parameters of the polynomial regression with response surface analysis of emotional empathy (EE) and cognitive empathy (CE), predicting Autism-Spectrum Quotient score in autistic and non-autistic individuals. The response surface parameters (a1-a4) are shown with a1 representing the linear effect of total empathy, a2 - the curvilinear effect of total empathy, a3 - the linear effect of empathic disequilibrium, and a4 – the curvilinear effect of empathic disequilibrium.

**p* < 0.05, ***p* < 0.005, ****p* < 0.0005

Predicting systemizing.

**Polynomial regression with response surface parameters predicting Systemizing Quotient after age exclusion.**

|  | **Autistic individuals** | | | **Non-autistic individuals** | | |  |
| --- | --- | --- | --- | --- | --- | --- | --- |
| **Effect** | **Estimate (SE)** | **p-value** | **beta** | **Estimate (SE)** | **p-value** | **beta** |  |
| **CE** | 2.27 (1.70) | 0.18 | 0.08 | 0.70 (0.48) | 0.15 | 0.02 |  |
| **EE** | -2.30 (1.3) | 0.077 | -0.08 | -2.91 (0.5) | 9x10^-9^ | -0.09*** |  |
| **CE^2^** | 3.33 (0.81) | 0.00004 | 0.25*** | 2.535 (0.45) | 2x10^-8^ | 0.1*** |  |
| **EE^2^** | 1.3 (0.51) | 0.01 | 0.09* | 1.2 (0.43) | 0.0049 | 0.05** |  |
| **CE x EE** | -0.525 (0.79) | 0.51 | -0.03 | -1.1 (0.51) | 0.031 | -0.04* |  |
| **Response surface parameters** | | | | | | | **Group Comparison (p-value)** |
| **a1** | 0.03 (1.875) | 0.99 | 0.006 | -2.21 (0.54) | 0.00005 | -0.07*** | 0.26 |
| **a2** | 4.10 (0.84) | 0.000001 | 0.30*** | 2.64 (0.54) | 2x10^-7^ | 0.11*** | 0.14 |
| **a3** | 4.57 (2.365) | 0.05 | 0.16 | 3.6 (0.82) | 0.00001 | 0.11*** | 0.7 |
| **a4** | 5.15 (1.58) | 0.001 | 0.37** | 4.84 (1.03) | 3x10^-6^ | 0.19*** | 0.87 |

Parameters of polynomial regression with response surface analysis of emotional empathy (EE) and cognitive empathy (CE), predicting Systemizing-Quotient score in autistic and non-autistic individuals. The response surface parameters (a1-a4) are shown with a1 representing the linear effect of total empathy, a2 - the curvilinear effect of total empathy, a3 - the linear effect of empathic disequilibrium, and a4 – the curvilinear effect of empathic disequilibrium.

**p* < 0.05, ***p* < 0.005, ****p* < 0.0005

**SI References**

1. Baron-Cohen, S., Wheelwright, S., Skinner, R., Martin, J. & Clubley, E. The Autism-Spectrum Quotient (AQ): Evidence from Asperger Syndrome/High-Functioning Autism, Males and Females, Scientists and Mathematicians. *Journal of Autism and Developmental Disorders* **31**, 5-17 (2001).

2. Baron-Cohen, S. & Wheelwright, S. The Empathy Quotient: An Investigation of Adults with Asperger Syndrome or High Functioning Autism, and Normal Sex Differences. *Journal of Autism and Developmental Disorders* **34**, 163-175 (2004).

3. Shanock, L.R., Baran, B.E., Gentry, W.A., Pattison, S.C. & Heggestad, E.D. Polynomial Regression with Response Surface Analysis: A Powerful Approach for Examining Moderation and Overcoming Limitations of Difference Scores. *Journal of Business and Psychology* **25**, 543-554 (2010).

4. Edwards, J.R. & Parry, M.E. On the Use of Polynomial Regression Equations As An Alternative to Difference Scores in Organizational Research. *Academy of Management Journal* **36**, 1577-1613 (1993).
